# Stimulus-specific enhancement in mouse visual cortex requires GABA but not VIP-peptide release from VIP interneurons

**DOI:** 10.1152/jn.00463.2023

**Published:** 2024-05-22

**Authors:** Megumi Kaneko, Mahmood S. Hoseini, James A. Waschek, Michael P. Stryker

**Affiliations:** ^1^Department of Physiology and Kavli Institute For Fundamental Neuroscience, University of California San Francisco, San Francisco, California, United States; ^2^Semel Institute for Neuroscience and Human Behavior, Department of Psychiatry and Biobehavioral Sciences, David Geffen School of Medicine, University of California Los Angeles, Los Angeles, California, United States

**Keywords:** GABA, mouse, plasticity, vasoactive intestinal peptide, visual cortex

## Abstract

When adult mice are repeatedly exposed to a particular visual stimulus for as little as 1 h per day for several days while their visual cortex (V1) is in the high-gain state produced by locomotion, that specific stimulus elicits much stronger responses in V1 neurons for the following several weeks, even when measured in anesthetized animals. Such stimulus-specific enhancement (SSE) is not seen if locomotion is prevented. The effect of locomotion on cortical responses is mediated by vasoactive intestinal peptide (VIP) positive interneurons, which can release both the peptide and the inhibitory neurotransmitter GABA. Previous studies have examined the role of VIP-ergic interneurons, but none have distinguished the individual roles of peptide from GABA release. Here, we used genetic ablation to determine which of those molecules secreted by VIP-ergic neurons is responsible for SSE. SSE was not impaired by VIP deletion but was prevented by compromising release of GABA from VIP cells. This finding suggests that SSE may result from Hebbian mechanisms that remain present in adult V1.

**NEW & NOTEWORTHY** Many neurons package and release a peptide along with a conventional neurotransmitter. The conventional view is that such peptides exert late, slow effects on plasticity. We studied a form of cortical plasticity that depends on the activity of neurons that express both vasoactive intestinal peptide (VIP) and the inhibitory neurotransmitter GABA. GABA release accounted for their action on plasticity, with no effect of deleting the peptide on this phenomenon.

## Graphical abstract

**Figure:**
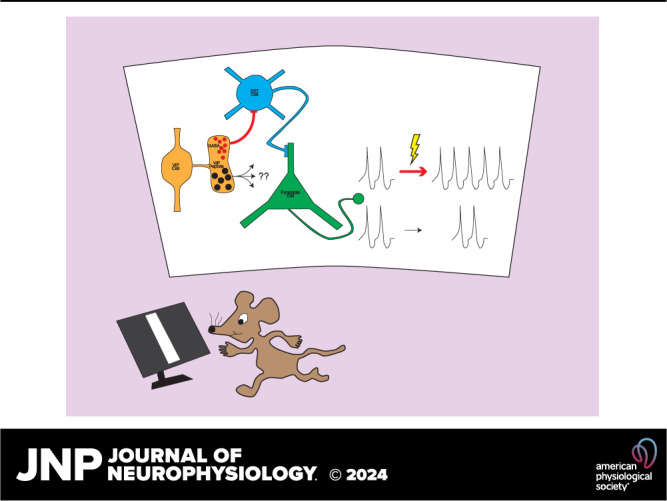

## INTRODUCTION

Recordings from alert mice revealed that behavioral state has a dramatic influence on the responses of neurons in the primary visual cortex (V1) (1). Locomotion, in particular, produced a highly reproducible increase in cortical responses while having little effect on the activity of neurons in the lateral geniculate nucleus (LGN). V1 neurons remained as selective during locomotion as when animals were still, and orientation selectivity was similar to that found in anesthetized animals (2).

Vasoactive intestinal peptide (VIP) positive interneurons play a crucial role in the increased responsiveness of V1 neurons during locomotion. When the VIP cells in a small region of V1 were destroyed, V1 responses remained normally selective but were no longer increased by locomotion (3, 4). When VIP cells in V1 were activated optogenetically in alert mice that were still, responses were increased just as if the animals were running (4) These findings showed that VIP-cell activity was both necessary and sufficient for the increase in selective V1 responses.

Locomotion also enhanced plasticity in a mouse model of recovery from deprivation amblyopia (5). When binocular vision was restored in mice that had been deprived of vision in one eye by unilateral lid suture for 4 mo beginning in early life, recovery assayed by intrinsic signal imaging was meager, less than halfway to normal levels of response. When these mice were instead given 4 h/day of visual stimulation during locomotion, the response to the stimuli viewed recovered to normal levels after 7–14 days. Microelectrode recordings confirmed the loss and restoration of visual responses (5). Blocking synaptic release from the VIP cells by their expression of tetanus toxin light chain prevented recovery of responses, whereas optogenetic stimulation of VIP cells in stationary mice produced recovery (3).

In normal adult mice, viewing a particular stimulus, such as a drifting bar or grating of a particular orientation, for 1 h/day while running on a polystyrene ball floating on an air stream produced a stimulus-specific enhancement (SSE) of V1 responses to that stimulus. Such SSE lasts for weeks and is evident both with intrinsic signal imaging through an intact skull, which is a completely noninvasive assay, and with single-cell measurements (6). Neurons selective for the stimulus viewed during locomotion increase their responses to it, whereas the responses of neurons that did not initially respond well to that stimulus are unchanged. Indeed, the resulting enhancement is proportional to the degree to which each neuron responded to the stimulus prior to the exposure during locomotion. Identical exposure to the stimulus without the opportunity for locomotion produced no enhancement (6).

These findings taken together suggest that some product of VIP-cell activity is responsible for SSE. VIP cells secrete the inhibitory neurotransmitter GABA, which appears principally to inhibit somatostatin-positive (SST) neurons and thereby disinhibit the excitatory neurons of V1. This disinhibition accounts for the increase in responses during locomotion (4). VIP cells also secrete the peptide VIP, the effects of which on the targeted cortical cells are not known. The present experiment seeks to determine which of these two VIP-cell secreted molecules is responsible for persistent SSE. We used both intrinsic signal imaging and microelectrode recording to study three groups of mice: those in which the peptide VIP was genetically deleted (*Vip^−^*^/−^); mice in which GABA release from VIP-cells was compromised by knocking out the vesicular GABA transporter (*VIP-Cre;Vgat^−^*^/−^); and control mice, including littermates of the *Vip^−^*^/−^ animals. Our conclusion is that SSE depends on GABA release from VIP cells but not on the secretion of the peptide VIP.

## MATERIALS AND METHODS

### Animals and Surgery

**Figure 1.**
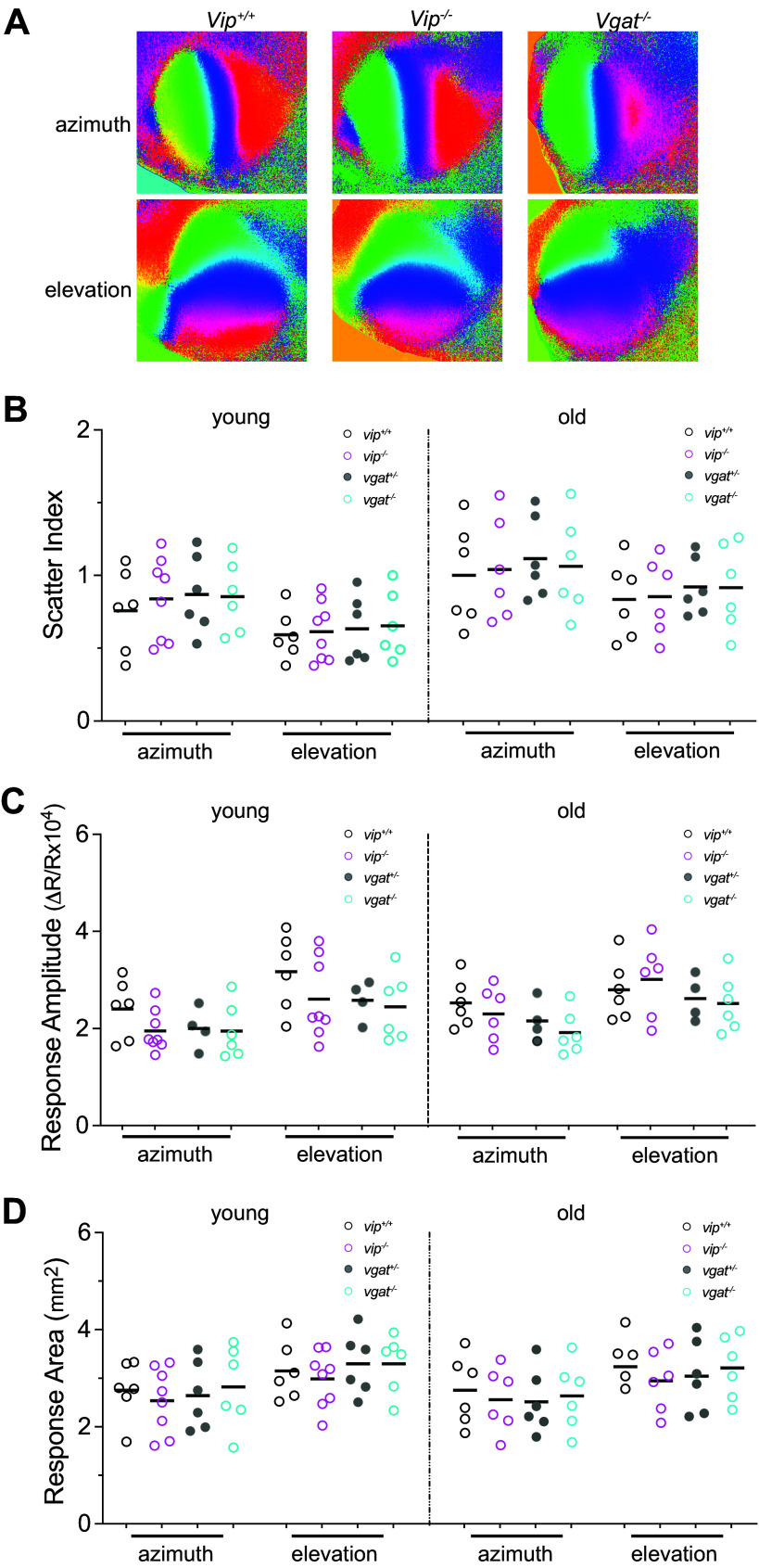
Baseline intrinsic signal responses in *Vip*^−/−^ and *Vgat*^−/−^ animals are indistinguishable from those in control animals. *A*: examples of retinotopic maps recorded with intrinsic signal imaging, in *Vip^+/+^*, *Vip^−/−^*, and *Vgat^−/−^* mice at young adult stage [*postnatal day* (*P*)*120*–*P140*). Azimuth and elevation maps are generated from responses to drifting vertical bars and horizontal bars, respectively. *B*: map scatter index. *C*: response amplitude. *D*: response area (mm^2^). *B–D*, *left*: young adults (*P120*–*P140*), and *B–D* (*right*): old mice (*P210*–*P240*). Data are statistically indistinguishable between *Vip^+/−^*, *Vip^−/−^*, *Vgat^+/−^*, and *Vgat^−/−^* animals, both in young and old adults.

**Figure 2.**
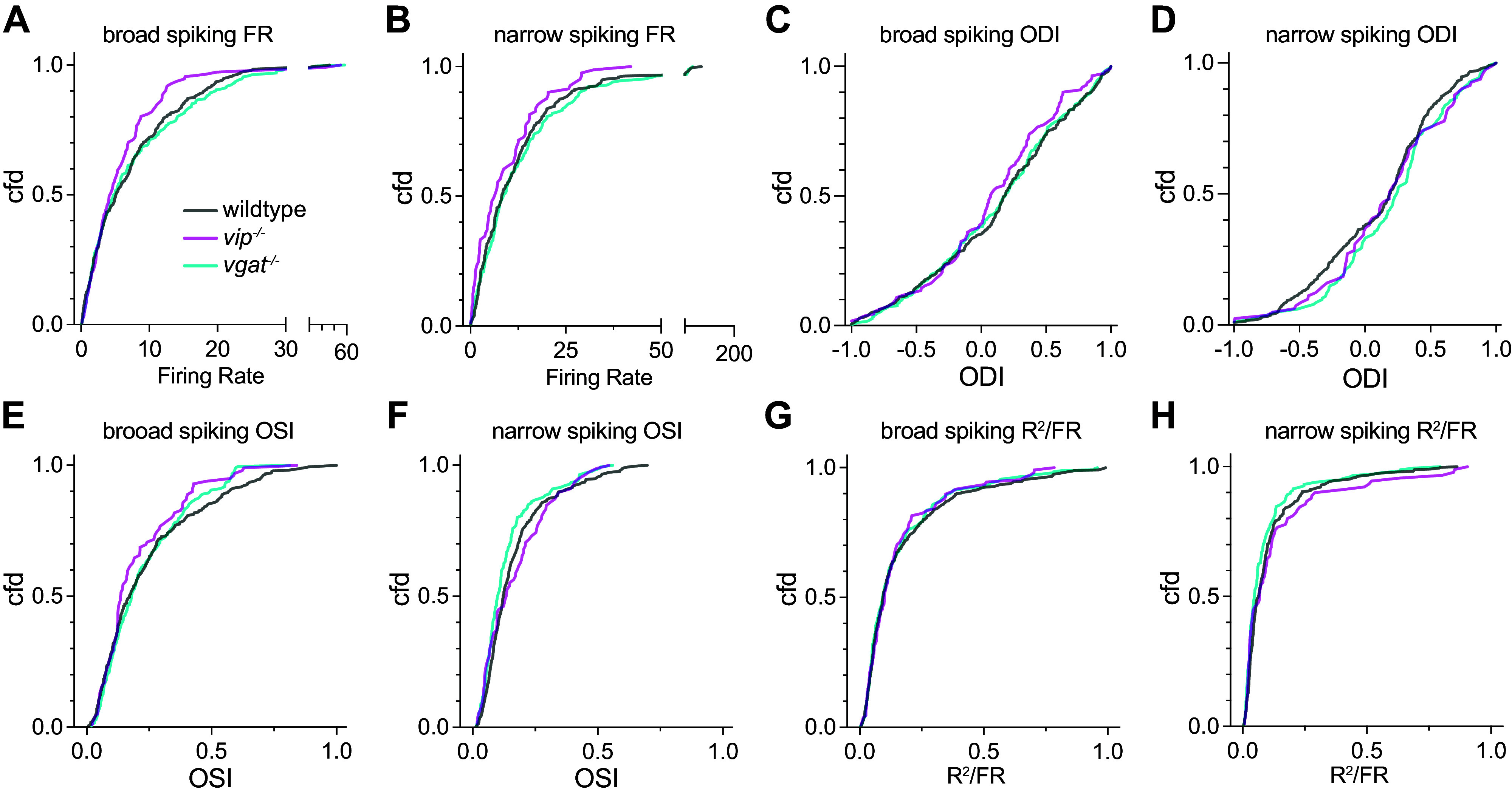
Response properties in individual neurons in visual cortex (V1) examined by single-unit electrophysiological recording. *A* and *B*: cumulative frequency distribution of firing rate (FR) of broad-spiking cells (*A*) and narrow-spiking cells (*B*) in response to contralateral-eye stimulation during still condition. *C* and *D*: cumulative frequency distribution of ocular dominance index in broad-spiking cells (*C*) and narrow-spiking cells (*D*). *E* and *F:* cumulative frequency distribution of orientation selectivity index in broad-spiking cells (*E*) and narrow-spiking cells (*F*). *G* and *H*: cumulative frequency distribution of a measure of response variability. Data from wild-type (WT), *Vip^−/−^*, and vasoactive intestinal peptide (VIP)-*Vgat^−/−^* animals are presented in gray, magenta, and cyan lines, respectively. Sample size: WT, broad-spiking: 374, narrow-spiking: 294; *Vip^−/−^*, broad-spiking: 111, narrow-spiking: 81; VIP-*Vgat^−/−^* broad-spiking: 261, narrow-spiking: 166. ODI, ocular dominance; OSI, orientation tuning.

**Figure 3.**
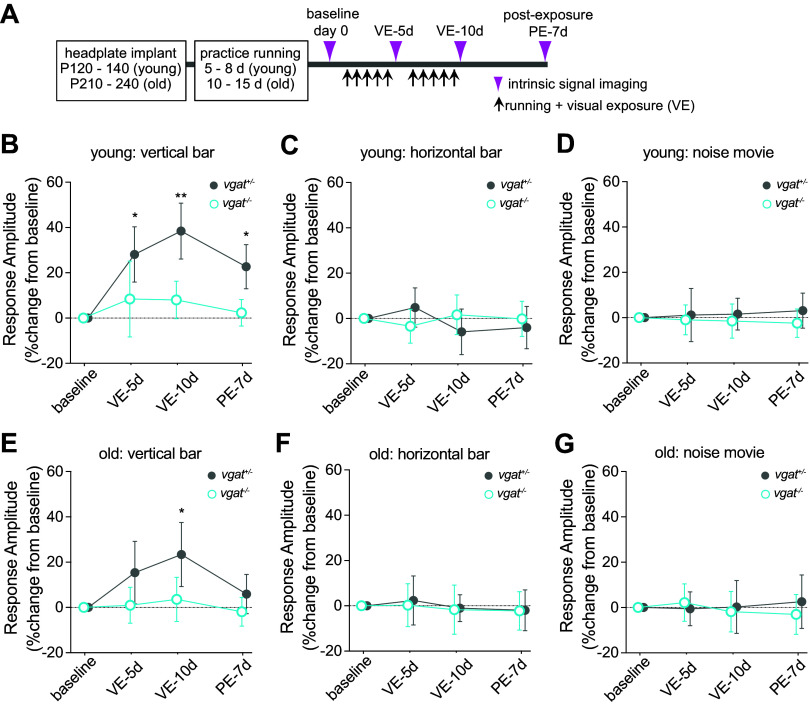
Stimulus-specific response enhancement is impaired in mice with Vgat loss-of-function. *A*: experimental schedule. *B* and *D*: change in response magnitude in young adult mice to exposed stimuli (*A*) and unexposed stimuli (horizontal bars, *B*; contrast-modulated noise movie, *C*) in control (VIP-*Vgat^+/−^*, filled gray circles, *n* = 5) and experimental animals (VIP-*Vgat^−/−^*, open cyan circles, *n* = 6). *E*–*G*: change in response magnitude in old animals to exposed stimuli (vertical bars, *E*) and unexposed stimuli (horizontal bars, *F*; contrast-modulated noise movie: *G*) in vasoactive intestinal peptide (VIP)-*Vgat^+/-^* control animals (gray filled circles, *n* = 5) and in VIP-*Vgat^−/−^* experimental animals (open cyan circles, *n* = 6). Response amplitude is expressed as percent change from baseline (100 × [post − baseline]/baseline). Error bars represent means ± SD. **P < 0.01, *P < 0.05 significant change from baseline response amplitude; two-way repeated-measures ANOVA followed by multiple comparisons with Bonferroni corrections. PE-7d, 7-day post-exposure; VE-5d, 5-day visual exposure; VE-10d, 10-day visual exposure.

**Figure 4.**
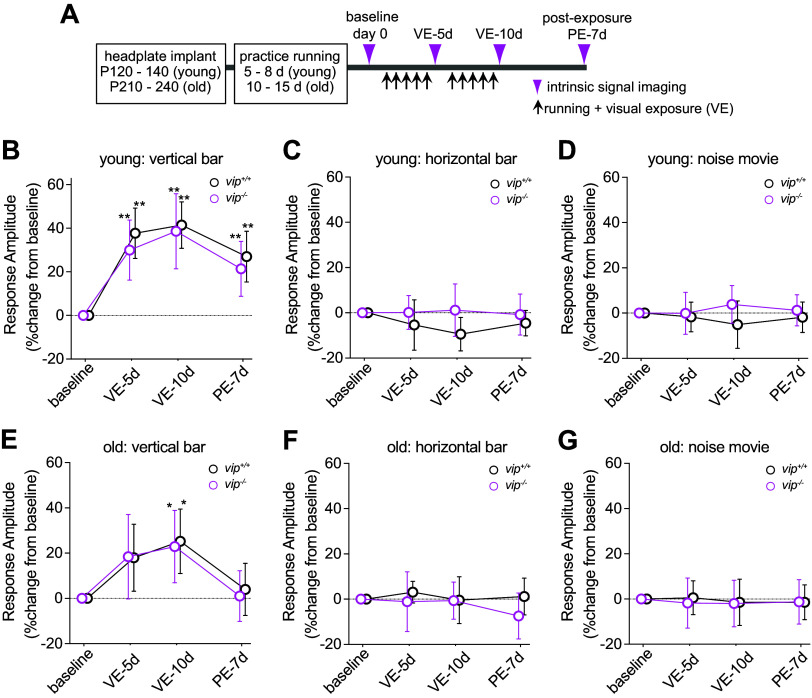
Stimulus-specific enhancement in vasoactive intestinal peptide (VIP) null mice is indistinguishable from that in Vip^+/+^ control mice. *A*: experimental schedule. *B*–*D*: change in response magnitude in young adult animals to exposed stimuli (vertical bars: *B*) and two kind of unexposed stimuli (horizontal bars: *C* and contrast-modulated noise movie: *D*) in *Vip^+/+^* control animals (black, *n* = 6) and in *Vip^−/−^* mice (magenta, *n* = 6). *E*–*G*: change in response magnitude in old animals to exposed stimuli (vertical bars: *E*) and unexposed stimuli (horizontal bars: *F* and contrast-modulated noise movie: *G*) in *Vip^+/+^* control animals (black *n* = 6) and in *Vip^−/−^* mice (magenta, *n* = 8). Response amplitude is expressed as percent change from baseline (100 × [post − baseline]/baseline). Error bars represent means ± SD. **P < 0.01, *P < 0.05 significant change from baseline response amplitude in each genotype group. PE-7d, 7-day post-exposure; VE-5d, 5-day visual exposure; VE-10d, 10-day visual exposure.

All procedures were approved by the Institutional Animal Care and Use Committee of University of California San Francisco. C57BL/6J (JAX 00664) wild type (WT), *VIP-ires-Cre* knock-in mice (*Vip^tm1(cre)Zjh^*/J; Catalog No. 010908) (7), and *Vgat^flox/flox^* (Catalog No. 012897) breeders were purchased from Jackson Laboratory (Bar Harbor, ME) and bred as needed, and animals of either sex were used. VIP-null mice were generated as previously described (8) and maintained by Dr. Waschek’s laboratory at University of California Los Angeles. VIP-null (*Vip^−^*^/−^) and mice wild type at the *VIP* locus (*Vip^+/+^*) were produced by crossing a *Vip^+/−^* female with a *Vip^+/−^* male for the experiments on SSE using intrinsic signal imaging shown in Figs. 1, 3, and 4. For the microelectrode recording experiments shown in Fig. 2, C57BL/6J mice were purchased from Jax and bred for use as wild-type (*Vip^+/+^*) control animals.

A custom stainless steel plate for head fixation was attached to the skull with dental acrylic under isoflurane anesthesia. The exposed surface of the skull was covered with a thin coat of nitrocellulose (New-Skin, Medtech Products Inc., NY) to prevent desiccation, reactive cell growth, and destruction of the bone structure. Animals were given a subcutaneous injection of an NSAID carprofen (5 mg/kg) just before and one day after the surgery. All mice were housed in groups of 4–5 and kept under standard conditions (12-h light/12-h dark cycle, with free access to food and water before the surgery, between recordings, and following daily running on the treadmill).

Because stimulus-specific enhancement declines with advanced age in mice, we studied separate groups of young adult [*postnatal day* (*P*)*120*–*P140*] and old (*P210*–*P240*) mice. SSE was studied in three animals of each age, genotype, and sex (except for 5 *Vip*^−/−^ and 2 *Vgat^+/−^* females), and results were similar between the sexes, although the numbers do not permit strong statistical comparison.

### Daily Exposure to Visual Stimuli during Locomotion

Each animal was acclimated to the running setting by the experimenter’s handling and being placed on the polystyrene ball floating on air for ∼15 min a day for 5–8 days in young animals or 10–15 days in old animals. Two to 3 days after measurement of baseline intrinsic-signal responses, animals were allowed to run on a 20-cm polystyrene ball for 60 min/day for 10 days, while viewing a 2° × 60° vertical bar drifting horizontally in both directions, presented on a 30 × 40 cm LCD monitor placed 25 cm from animal’s right eye in a dark room, as described previously (6).

### Intrinsic Signal Optical Imaging

Repeated optical imaging of intrinsic signals was performed through the intact skull as described previously (9). Five to 7 days after the headplate implantation, the first imaging of intrinsic signals was performed to measure baseline responses. The mouse was anesthetized with isoflurane (3% for induction and 0.7% during recording), supplemented with intramuscular injection of chlorprothixene chloride (2 µg/g body wt), and images were recorded transcranially through the window of the implanted headplate. Intrinsic signal images were obtained with a Dalsa 1M30 CCD camera (Dalsa, Waterloo, ON, Canada) with a 135 mm × 50 mm tandem lens (Nikon Inc., Melville, NY) and red interference filter (610 ± 10 nm). Frames were acquired at a rate of 30 fps, temporally binned by 4 frames, and stored as 512 × 512 pixel images after binning the 1,024 × 1,024 camera pixels by 2 × 2 pixels spatially. Responses in each mouse were measured with three kinds of visual stimuli, *1*) horizontal bars drifting upward or downward, *2*) vertical bars drifting leftward or rightward, and *3*) the contrast-modulated noise movie. They were generated in Matlab using Psychophysics Toolbox extensions (10, 11), and displayed on an LCD monitor (30 × 40 cm, 600 × 800 pixels, 60-Hz refresh rate) placed 25 cm from the mouse, spanning ∼60° (height) × ∼77° (width) of visual space. The drifting bar was the full length of the monitor and 2° wide, and it moved continuously and periodically (12). The contrast-modulated Gaussian noise movie consisted of the Fourier-inversion of a randomly generated spatiotemporal spectrum with low-pass spatial and temporal cutoffs applied at 0.05 cpd and 4 Hz, respectively (2). To provide contrast modulation, the movie was multiplied by a sinusoid with a 10-s period. Movies were generated at 60 × 60 pixels and then smoothly interpolated by the video card to 480 × 480 to appear ∼60° (height) × ∼60° (width) on the monitor and played at 30 frames/s. Each recording took 240 s and was repeated for at least six measurements per animal. During the daily session of running + visual exposure (VE), animals were exposed to only drifting vertical bars as described earlier.

### Analysis of Intrinsic Signal Images

The region of interest (ROI) within V1 was selected on the response magnitude map evoked by visual stimulation. First, the map was smoothed to reduce pixel shot noise by low-pass filtering using a uniform kernel of 5 × 5 pixels. The background area was selected from the area covering ∼150 × 150 pixels outside of V1. The ROI was selected by thresholding at 300% above the average background amplitude and the response amplitude was then calculated as the average amplitude of pixels within the ROI.

### Microelectrode Recording

Extracellular recording was carried out in awake, head-fixed mice as in the work by Hoseini et al. (13) that were free to run on the floating polystyrene ball. On the day of recording, the animal was anesthetized and a craniotomy of about 1–2 mm in diameter was made above the binocular zone of V1 (identified by intrinsic signal imaging). This small opening was large enough to allow for insertion of a 1.1-mm long double-shank 128-channel electrode fabricated by the Masmanidis laboratory (14) and assembled by the Litke laboratory (University of California Santa Cruz). The electrode was placed at an angle of 20°–40° to the cortical surface and inserted to a depth of 500–1,000 μm. Recordings were started an hour after implantation. For each animal, the electrode was inserted not more than twice.

Visual stimuli were displayed on an LCD monitor (Dell, 30 ×40 cm, 60 Hz refresh rate, 32 cd/m^2^ mean luminance) placed 25 cm from the mouse (−20° to +40° elevation). Drifting sinusoidal gratings at 12 evenly spaced directions (30 steps, 1.5-s duration, 0.04 cycles/degree, and 1 Hz temporal frequency) were generated and presented in random sequence using the MATLAB Psychophysics Toolbox followed by 1.5-s blank period of uniform 50% gray.

Movement signals of locomotion from optical mice tracking the polystyrene ball were acquired in an event-driven mode at up to 300 Hz, and integrated at 100-ms intervals and then converted to the net physical displacement of the top surface of the ball. A mouse was said to be running on a single trial if its average speed for the first 500 ms of the trial fell above a threshold, found individually for each mouse (1–3 cm/s), depending on the noise levels of the mouse tracker. Data acquisition was performed using an Intan Technologies RHD2000-Series Amplifier Evaluation System, sampled at 20 kHz; recording was triggered by a TTL pulse at the moment visual stimulation began.

Single units were identified using MountainSort (15), which allows for automated spike sorting of the data acquired using 128-site electrodes. Following manual curation, typical yields ranged between 50 and 130 isolated single units. Average waveforms of isolated single units were used to calculate three parameters by which cells were classified into narrow- or broad-spiking.

Orientation tuning curves from which response parameters were calculated were fitted as a mixture of two Gaussians of individually variable height and width separated by 180° for visual stimulus space.

### Statistical Analyses

Data are presented as means ± SE or means ± SD, unless otherwise indicated. Sample numbers (*n*) are indicated in the figure legends or in results. Comparison between groups with multiple times was performed using a two-way repeated-measures analysis of variance (RM-ANOVA) followed by Bonferroni’s post hoc test. Three groups or more without time series were compared by a one-way ANOVA with Bonferroni’s post hoc test or Kruskal–Wallis test with Dunn’s post hoc test. Statistical analyses were performed using PRISM (GraphPad Software, CA) or Matlab (MathWorks, MA). The difference was considered significant at *P* < 0.05.

## RESULTS

### Characterization of Visual Responses in V1 of VIP Null Animals and Mice with Disrupted Vgat in VIP Cells Using Intrinsic Signal Imaging

VIP-null (*Vip^−^*^/−^) mice and mice wild type at the VIP locus (*Vip^+/+^*) were produced by crossing a *Vip^+/−^* female with a *Vip^+/−^* male for the experiments on SSE using intrinsic-signal imaging shown in Figs. 1, 3, and 4. The VIP expression in the VIP-null (*Vip*^−/−^) mouse line used in this study is below detectable levels in whole brain homogenate, as is the mRNA for VIP (16). To examine the role of vesicular GABA release from VIP cells in SSE, we used the conditional vesicular GABA transporter (*Vgat*) loss-of-function mice, and generated animals for study by crossing *Vgat^flox/flox^* mice with *VIP-Cre::Vgat^flox/+^* mice. The products of this breeding strategy, *Vgat^+/−^* and *Vgat^−^*^/−^ mice were used as control and experimental animals, respectively. Vgat is required for the loading of GABA into synaptic vesicles and therefore essential for the normal synaptic release of GABA. It is well established that synaptic GABA release is absent in cells of the floxed *Vgat* mouse line that express Cre recombinase (17–24).

We first examined visual cortical responsiveness at a global level using intrinsic signal imaging (12) under isoflurane anesthesia in young adult mice (*P120*–*P140*) and in old mice (*P210*–*P240*) (Fig. 1). Examples of cortical retinotopic maps in response to vertical bars drifting right-left (azimuth maps) and to horizontal bars drifting up-down (elevation maps) are shown in Fig. 1*A*. The gross polarity of the retinotopic map in V1 was largely normal in both *Vip^−^*^/−^ and *Vgat^−^*^/−^ mice. To compare the maps quantitatively, we computed the “map scatter” by calculating the differences between the phase values of the individual pixels within the visual area to those of their near neighbors, as previously described (25). For maps with high quality and strong visual response, these phase differences should be quite small due to the smooth progression of V1 topography. For the azimuth maps, the map scatter in *Vip^+/+^* mice was 0.75 ± 0.28° (young, *n* = 6) and 1.0 ± 0.35° (old, *n* = 6), in *Vip^−^*^/−^ mice 0.84 ± 0.29° (young, *n* = 8) and 1.04 ± 0.35° (old, *n* = 6), in *Vgat^+/−^* mice 0.87 ± 0.27° (young, *n* = 6) and 1.12 ± 0.28° (old, *n* = 6), and in *Vgat^−^*^/−^ mice 0.85 ± 0.25° (young, *n* = 6) and 1.06 ± 0.33° (old, *n* = 6); all statistically insignificant from each other (Fig. 1*B*) [one-way ANOVA: young: *F*(2,17) = 0.216, *P* = 0.808; old: *F*(2,15) = 0.050, *P* = 0.951]. Scatter index in elevation maps are similarly comparable among *Vip^+/+^*, *Vip^−^*^/−^, *Vgat^+/−^*, and *Vgat^−^*^/−^ animals. *Vip^+/+^*: 0.59 ± 0.17° (young), 0.83 ± 0.27° (old); *Vip^−^*^/−^: 0.62 ± 0.20° (young), 0.85 ± 0.27° (old); *Vgat^+/^*^−^: 0.63 ± 0.23° (young), 0.92 ± 0.20° (old); *Vgat^−^*^/−^: 0.66 ± 0.23° (young), 0.92 ± 0.30° (old) (Fig. 1*B*) [one-way ANOVA: young: *F*(2,17) = 0.150, *P* = 0.862; old: *F*(2,15) = 0.132, *P* = 0.878].

Cortical responses to visual stimulation in *Vip^−^*^/−^ mice and *Vgat^−^*^/−^ mice were not statistically different in amplitude from those in *Vip^+/+^* and *Vgat^+/−^* animals, respectively, at a global level as measured by intrinsic signal optical imaging (Fig. 1*C*). The average amplitude of azimuth maps in *Vip^+/+^* mice: 2.4 ± 0.61 deltaR/R (young), 2.53 ± 0.49 (old); in *Vip^−^*^/−^ mice: 1.95 ± 0.42 (young), 2.3 ± 0.56 (old); in *Vgat^+/−^* mice: 2.00 ± 0.43 (young), 2.16 ± 0.42 (old); in *Vgat^−^*^/−^ mice: 1.95 ± 0.42 (young), 1.92 ± 0.45 (old) [one-way ANOVA: young: *F*(2,17) = 1.581, *P* = 0.235; old: *F*(2,15) = 2.269, *P* = 0.138]. The average amplitude of elevation maps in *Vip^+/+^* mice: 3.17 ± 0.78 (young), 2.85 ± 0.61 (old); in *Vip^−^*^/−^ mice: 2.61 ± 0.82 (young), 3.01 ± 0.78 (old); in *Vip^+/−^* mice: 2.58 ± 0.41 (young), 2.62 ± 0.46 (old); in *Vgat*^−/−^ mice: 2.45 ± 0.69 (young), 2.52 ± 0.58 (old) [one-way ANOVA: young: *F*(2,17) = 1.478, *P* = 0.256; old: *F*(2,15) = 0.838, *P* = 0.452].

Similarly, the response areas in *Vip^−^*^/−^ mice and *Vgat^−^*^/−^ mice were indistinguishable from those in respective control animals, for both azimuth and elevation maps in both young age and old adult mice (Fig. 1*D*) [one-way ANOVA: azimuth maps: young: *F*(2,17) = 0.315, *P* = 0.734, old: *F*(2,15) = 0.116, *P* = 0.891; elevation maps: young: *F*(2,17) = 0.486, *P* = 0.623, old: *F*(2,15) = 0.389, *P* = 0.684].

These observations indicate that, with normal visual experience, visual cortical function in *Vip^−^*^/−^ mice and *Vgat^−^*^/−^ mice develop without detectable impairment in the measures we evaluated.

### Response Properties and Selectivity of Single Neurons

For the microelectrode recording experiments shown in Fig. 2, C57BL/6J mice were purchased from Jax and bred for use as wild-type (WT) control animals. Microelectrode recordings made in alert *Vip^−^*^/−^ and *VIP-Cre::Vgat^−^*^/−^ mice were compared with those made in WT mice in terms of firing rates to gratings, indices of ocular dominance and orientation selectivity, and variability in response. Head-fixed mice viewing a video monitor were free to stand or run on a polystyrene ball floating on air while 2-shank silicon probes with 128 recording sites were lowered into the binocular zone of V1. After spike sorting, the waveforms of isolated single units were classified as broad-spiking (putatively excitatory) or narrow-spiking (putatively inhibitory). Mice in these experiments had no prior practice on the polystyrene ball and were therefore mostly still, rather than walking or running, during recording.

Figure 2 shows cumulative frequency distributions for firing rate (FR), ocular dominance (ODI), and orientation tuning (OSI). Because firing rates are strongly influenced by locomotion, we compare them in Fig. 2, *A* and *B* only during comparable still periods. Firing rates elicited by stimulation through the contralateral eye of both broad- and narrow-spiking neurons were essentially identical in *Vgat^−^*^/−^ and WT mice but both appeared slightly lower in *Vip^−^*^/−^ mice, although not statistically significant revealed by Kruskal–Wallis tests (Fig. 2, *A* and *B*) [broad-spiking: *H*(2) = 1.835, *P* = 0.399; narrow-spiking: *H*(2) = 5.259, *P* = 0.072]. The ocular dominance distributions of both broad- and narrow-spiking neurons were nearly identical in the three groups of mice (Fig. 2, *C* and *D*) [broad-spiking: *F*(2,743) = 0.516, *P* = 0.597; narrow-spiking: *F*(2,544) = 2.702, *P* = 0.068; one-way ANOVA]. Also, the distributions of orientation selectivity as measured through the contralateral eye were not significantly different in the three groups [broad-spiking: *F*(2,743) = 1.927, *P* = 0.146; narrow-spiking: *F*(2,544) = 2.89, *P* = 0.059; one-way ANOVA]. We constructed a measure of response variability for each neuron analogous to coefficient of variation by dividing the “goodness of fit” of the orientation tuning curve by the neuron’s firing rate at its optimal orientation (*R*^2^/FR, Fig. 2, *G* and *H*). These distributions of response variability, which exclude periods of locomotion, were not significantly different among three groups of mice for both broad- and narrow-spiking neurons (Fig. 2, *G* and *H*) [broad-spiking: *H*(2) = 0.233, *P* = 0.890; narrow-spiking: *H*(2) = 5.511, *P* = 0.064; Kruskal–Wallis tests].

Before the critical period of susceptibility to the effects of monocular visual deprivation, at around 4 wk of age, the preferred stimulus orientation of individual V1 neurons driven through one eye is only randomly related to the preferred orientation measured through the fellow eye (26). After visual experience during the critical period, orientation selectivity measured through the two eyes becomes similar. One measure of the normal development of visual cortical organization, therefore, is the similarity of preferred orientation between orientation-selective neurons as assessed through the two eyes. Cells were excluded from this analysis if FR < 1 Hz or OSI < 0.2 when driven through either eye or if their receptive fields were not located in the binocular zone within 15° of the vertical meridian, resulting in sample sizes as follows: WT: 165, *Vip^−^*^/−^: 46, and *Vgat^−^*^/−^: 131. The median differences in preferred orientation between two eyes for the two experimental groups of mice were similar to and statistically indistinguishable from those of WT mice [medians and interquartile range: WT 24° ± 16°, *Vip^−^*^/−^ 26° ± 22°, *Vgat^−^*^/−^ 29° ± 23°; *H*(2) = 2.356, *P* = 0.308, Kruskal–Wallis test].

### Vesicular GABA Release from VIP Cells Is Required for Stimulus-Specific Response Enhancement

After confirming that cortical responsiveness to visual stimuli in *Vgat^−^*^/−^ and *Vip^−^*^/−^ mice is largely normal at a global level or at a single-neuron level, we next tested whether disruption of GABAergic transmission from VIP cells affects SSE. Figure 3*A* shows the experimental schedule. Each animal was acclimated to the experimental apparatus by handling and being placed on the polystyrene ball floating on air in a dimly lighted room with no moving visual stimuli. Young adult mice were acclimated for ∼15 min a day for 5–8 days, and old mice for 10–15 days. Baseline intrinsic signal responses were then measured to three different, high-contrast visual stimuli: a drifting horizontal bar, a drifting vertical bar, and contrast-modulated noise movie. Starting 2–3 days later, animals were allowed to run on polystyrene balls while viewing the drifting vertical bar for 60 min per day for 10 days, as described previously (6). Our earlier experiments had shown that the effect of 1 h/day of exposure produced saturating enhancement effects; doubling the exposure time per day did not increase or accelerate SSE (6). To track the change in visual cortical responses to these visual patterns, we repeated intrinsic signal recordings after 5 days and 10 days of visual exposure (VE); and additionally 1 wk after stopping the VE sessions (postexposure PE-7d) (Fig. 3*A*).

Young adult *Vgat^+/−^* control mice showed a significant increase from baseline response level in response only to the vertical bar that the animal viewed during daily running (28.4 ± 14.1% above baseline after 5-day VE, gray circles in Fig. 3*B*), whereas responses to other stimuli were unchanged (horizontal bar: −5.8 ± 11.6%, Fig. 3*C*; noise movie: 1.0 ± 13.6%, Fig. 3*D*). This enhancement was slightly strengthened after 10-day VE (38.7 ± 14.2%). The response amplitude to the exposed stimulus stayed significantly elevated for at least 7 days after terminating sessions of running + VE (postexposure PE-7d) (22.6 ± 11.3%) (Fig. 3*B*). In contrast to the heterozygous control animals, *Vgat^−^*^/−^ mice failed to show enhancement of responses to the stimulus that the animal viewed during daily running (cyan circles in Fig. 3*B*; 8.4 ± 16.7% after VE-5d, 8.1 ± 8.3% after VE-10d).

Compared with young control animals, response enhancement to the exposed stimulus in old *Vgat*^+/−^ control mice was smaller and significantly apparent only after 10 days of running + VE (14.3 ± 16.8% at VE-5d, 20.1 ± 17.6% at VE-10d) (Fig. 3*E*). This small enhancement in old *Vgat*^+/−^ control mice did not persist 7 days postexposure (4.8 ± 9.6%; Fig. 3*E*). The current observations in the young heterozygous control mice are consistent with our previous report (6).

In contrast to the *Vgat*^+/−^ control animals, *Vgat^−^*^/−^ experimental mice failed to show enhancement of responses to the stimulus that the animal viewed during daily running. This impairment was observed both in young adult mice (VE-5d: 8.4 ± 16.7%, VE-10d: 8.1 ± 8.3%, PE-7d: 2.4 ± 5.9; Fig. 3*B*) and in old mice (VE-5d: 1.0 ± 7.9%, VE-10d: 3.6 ± 9.7%, PE-7d: −1.9 ± 6.4%; Fig. 3*E*).

In young animals as shown in Fig. 3*B*, a two-way repeated-measures analysis of variance (RM-ANOVA) revealed that there was a statistically significant interaction between the effects of time and genotype [*F*(3,27) = 6.789, *P* = 0.0015]. Simple main effects analysis showed that both time and genotype had a statistically significant effect (*P* < 0.0001 for time, *P* = 0.0024 for genotype). Post hoc multiple comparisons indicated that *Vgat*^+/−^ control mice had statistically significant increase in response amplitude after VE-5d, VE-10d, and PE-7d compared with baseline, whereas responses in *Vgat*^+/−^ experimental mice did not change over this time course (Fig. 3*B*). Accordingly, response changes at VE-10d and PE-7d are statistically different between *Vgat*^+/−^ control and Vgat^−/−^ mice (*P* = 0.21 at VE-5d, *P* = 0.0096 at VE-10d, *P* = 0.023 at PE-7d).

Also in old animals as shown in Fig. 3*D*, there was a statistically significant interaction between the effects of time and genotype [*F*(3,27) = 3.14, *P* = 0.042]. Simple main effects analysis showed that both time and genotype had statistically significant effects (*P* = 0.0054 for time, *P* = 0.0133 for genotype). Post hoc multiple comparisons indicated statistically significant increase from baseline in response amplitude at VE-10d in *Vgat*^+/−^ control mice, whereas *Vgat^−^*^/−^ mice showed no change from baseline.

### VIP Is Not Required for Stimulus-Specific Response Enhancement

Mice deficient in VIP (*Vip^−^*^/−^) have previously been reported to exhibit various neurological, developmental, and/or behavioral deficits (8, 27–30). We first examined V1 visual responses in *Vip^−^*^/−^ mice at a global level, using intrinsic-signal imaging to measure retinotopic organization and response magnitude, as shown in Fig. 1. We also performed microelectrode recording from individual neurons in V1 to assess their response magnitude, orientation tuning, and ocular dominance, as shown in Fig. 2. After confirming largely normal visual cortical responsiveness of *Vip^−^*^/−^ mice, we next tested whether the absence of VIP affects SSE. Figure 4*A* shows the experimental schedule. Consistent with our previous report (6) and with our findings aforementioned in *Vgat^+/+^* control mice, young adult *Vip^+/+^* control mice showed a significant increase in cortical responses to the exposed stimulus (vertical bar) after daily visual exposure while running (VE-5d: 37.7 ± 11.6%; VE-10d: 41.5 ± 10.7%), which persisted at PE-7d (27 ± 11.6%) (Fig. 4*B*), while no significant change to unexposed stimuli was detected (horizontal bar VE-5d: −5.4 ± 11.2%, VE-10d: −9.5 ± 7.4%, PE-7d: −4.6 ± 5.6%, Fig. 4*C*; noise movie VE-5d: −1.7 ± 6.5%, VE-10d: −5.1 ± 10.5%, PE-7d: −1.9 ± 6.7%, Fig. 4*D*). Similarly, a small but statistically significant increase in response to the exposed stimulus was observed in old *Vip^+/+^* control animals after 10 days of daily running + VE (VE-5d: 17.9 ± 14.9%, VE-10d: 25.3 ± 14.3%), which did not persist postexposure (4.0 ± 11.5%, Fig. 4*E*).

In *Vip^−^*^/−^ mice, the increase in response magnitude to the exposed stimulus during VE was statistically indistinguishable from that in WT control mice, both in young adults (VE-5d: 29.9 ± 13.8%, VE-10d: 38.6 ± 17.2%) and in old animals (VE-5d: 18.4 ± 18.7%; VE-10d: 22.9 ± 16.0%). Persistence of enhancement was also similar to WT control animals both in young adults and old mice (VE-7d: 21.4 ± 12.6% and 1.0 ± 11.2%, respectively) (Fig. 4, *B* and *E*).

In young animals as shown in Fig. 4*B*, a two-way RM-ANOVA revealed that there was not a statistically significant interaction between the effects of time and genotype [*F*(3,36) = 0.4997, *P* = 0.685]. Simple main effects analysis showed that time had a statistically significant effect (*P* < 0.001), whereas genotype did not have a significant effect (*P* = 0.403). Post hoc tests showed that both *Vip^+/+^* control and *Vip^−^*^/−^ mice had statistically significant response enhancement to exposed stimuli at VE-5d, VE-10d, and PE-7d compared with baseline, whereas no statistically significant differences were detected between these two genotypes.

Similarly in old animals as shown in Fig. 4*E*, the interaction between the time and genotype effects [*F*(3,30) = 0.103, *P* = 0.958] was not statistically significant [*F*(3,30) = 0.103, *P* = 0.958]. Simple main effects analysis showed statistically significant time effect (*P* < 0.001) but insignificant genotype effect (*P* = 0.8387). Post hoc tests demonstrated that both *Vip^+/+^* control mice and *Vip^−^*^/−^ experimental mice had statistically significant response enhancement to the exposed stimuli only at VE-10d.

## DISCUSSION

### Development and Function of Visual Cortex with Mutant VIP Cells

The present experiments reveal that visual cortical organization developed normally when VIP cells were compromised either by the deletion of the VIP peptide or by the impairment of GABA release. The maps of the visual cortex obtained by intrinsic signal imaging had normal orientation and signal-to-noise in the two genetically compromised mouse strains, and the magnitudes of visual responses measured in intrinsic signal imaging were also like those in WT mice. In addition, microelectrode recordings of the responses of single neurons revealed highly similar distributions of all measures of visual response that were obtained. We conclude from this that VIP cells need not release either GABA or VIP peptide for the normal development of these functions of the visual cortex.

This is somewhat surprising in light of previous studies that reported various developmental disruption of VIP interneurons in the cortex perturb normal sensory processing and activity patterns in adult mice (31–33), though subtle effects on cortical responses may not have been evident in the present measurements. The extent to which VIP release, GABA release, or both, are reduced by developmental disruptions such as VIP-cell specific ErbB4 (31) or MeCP2 (32) or SCN1A (34) mutation are not clear.

It is unlikely that loss of VIP or GABA release in the present study was incomplete. VIP expression was abolished to undetectable levels in the brain of the global VIP knockout (KO) mice used in our studies by immunohistochemistry (8) and more sensitive radioimmunoassay (16). Also, it is well established that synaptic GABA release is absent in cells of the floxed *Vgat* mouse line that express cre recombinase (18–24, 35, 36). In addition, we have confirmed loss of VGAT from presynaptic regions in long-term cultures of *vgat^−^*^/−^ cells (37) derived from mice in the same colony that we used in the current study.

### VIP Cells in Adult Plasticity

Recent studies have shown the role of VIP neurons in the state-dependent regulation of cortical activity (4, 38, 39). VIP neurons are activated during high arousal, behaviorally relevant input (40, 41), and locomotion (4, 40, 42). VIP neurons are considered to influence the local circuit partly through their inhibition of SST neurons (43–45). Inhibition of SST neurons by VIP neurons disinhibits local excitatory neurons, resulting in magnification of pyramidal neuron activity (40, 42, 44, 46–51).

We have previously shown that VIP neurons play critical roles through this disinhibition not only in instantaneous increase in V1 pyramidal neuron responses (4) but also in V1 plasticity following sensory manipulations such as monocular deprivation long after the critical period that was greatly suppressed by silencing synaptic transmission in VIP neurons (by viral vector-mediated expression of tetanus toxins) (3). We have demonstrated that locomotion is required for enhancement of response in V1 to a particular stimulus presented repeatedly (stimulus-specific enhancement, SSE) (6). Taken together, these results suggest that synaptic release from VIP neurons is critical for inducing SSE. VIP neurons release both GABA and VIP peptide. Our previous manipulations of VIP cells—ablation and tetanus toxin expression—similarly affect both products (3, 4). Studies from other laboratories have reported that disruptions of VIP neurons with a number of different manipulations (31, 32, 34, 47, 52, 53) result in dysregulation in cortical function, but none of these specifically alter GABAergic or VIP-ergic transmission. We therefore sought to eliminate them one at a time to assay their separate effects on SSE. Lacking a conditional knockout (KO) of VIP peptide, we were justified in interpreting results in V1 of the global knockout of VIP peptide by the fact that even VIP loss in all neurons (not just V1 interneurons) failed to affect SSE. A conditional knockout of the vesicular GABA transporter allowed us to delete it specifically in VIP cells.

### Relation to Other Reports of Similar Plasticity

Previous studies using evoked potential recordings through electrodes implanted in the visual cortex reported a rapid and persistent increase in layer 4 responses to repeated stimuli that were similar to SSE in magnitude and dependence on *N*-methyl-d-aspartate (NMDA)-receptor signaling (6, 54, 55). Single-unit recordings from adult mice in which chronic stereotrodes had been implanted into the visual cortex found that presentation of a contrast-reversing full-field grating for an hour induced a subsequent orientation-specific increase in response of putative excitatory cells if sleep were permitted and a decrease if the mice were subsequently sleep deprived (56). These evoked potential and single-unit findings differed from SSE in that they were found even in restrained animals and are therefore unlikely to depend on VIP cell activity.

Several factors may contribute to this difference. First, different techniques were used to measure responsiveness. Intrinsic signal imaging used in this study is completely noninvasive and has been shown to produce stable read-out of visual cortical responsiveness over weeks (5). In contrast, chronic visual evoked potential (VEP) and stereotrode recording require electrode implantation right into the brain tissue at the center of interest, which could induce inflammatory responses including reactive astrocytes. Such disruptions can result in an increase in excitability of pyramidal cells resulting from dysfunction in astrocytes for microenvironmental homeostasis and/or maintenance of GABAergic inhibition, reviewed by Robel and Sontheimer (57). This increased excitation-inhibition balance may induce abnormally high degree of plasticity in adult brain, reviewed by Bavelier et al. (58).

Second, the VEP studies focused on layer 4 (L4) and the single-unit studies were largely of the infragranular layers, whereas the present study’s intrinsic optical signals reflect activity in L2/3 more strongly than in L4, with a significant contribution from L4 (59). Perceptual learning has been shown to have different effects on L2/3 and L4 pyramidal neurons in mouse V1 (60). L2/3 neurons acquired a new response pattern suggestive of anticipatory response whereas L4 neurons did not. This change in L2/3 was accompanied by increased excitatory drive from top-down inputs. In rats, L2/3 excitatory cells in primary somatosensory cortex (S1) showed tonic activation pattern that was longer in duration and higher in magnitude than L4 cells during active tactile discrimination, presumably also resulting from the influence by top-down inputs (61). Similarly, after visual discrimination training in rhesus monkeys, sharpening of the orientation tuning curve was observed in supragranular layer but not in L4 (62).

In addition to laminar differences, intracortical microcircuits that participate in the enhancement of responses in L4 may be distinct from locomotion-induced response enhancement that we observed. Kaplan et al. (63) have implicated the activity of parvalbumin-expressing (PV) GABAergic cells in L4 enhancement. In contrast, as described earlier, a disinhibitory circuit comprised of VIP cells and SST cells plays a key role in control of the gain of visual responses and in facilitation of plasticity by locomotion in adult visual cortex, whereas PV neurons do not show consistent responses to locomotion (3, 4).

### VIP Neurons, Sleep, and Cortical Plasticity

The orientation-specific response potentiation (OSRP) observed in adult mouse visual cortex, which is similar to SSE, is blocked by disruption of sleep, especially of non-rapid eye movement (REM) sleep (56, 64). Although VIP-null mice did indeed exhibit altered sleep patterns when housed, like the mice in the present study, in a regular 12-h/12-h light-dark environment (8, 65), we show here that they have normal SSE. Although *VIP-Vgat^−^*^/−^ mice are known to have normal circadian rhythms (66, 67), sleep disruptions in these mice have not been reported, and such a disruption could conceivably account for the abnormal SSE we observed here in them.

### Conclusions

In the current study, we have demonstrated that compromising GABA release from VIP cells completely blocked SSE, whereas blocking the release of VIP peptide had no effect on this form of plasticity. The effects were consistent in both young adult and old adult mice. These findings suggest that the effect of VIP cell activity to inhibit the firing of the other neurons of the visual cortical circuit is responsible for SSE. Since VIP cell activity inhibits SST cells and thereby disinhibits the excitatory neurons, it allows them to respond more vigorously to the visual stimuli for which they are selective (43–45). SSE might then result from ordinary Hebbian plasticity. Stimulating with a bar of a particular orientation while the activities of the V1 neurons that respond to it are elevated should engage whatever Hebbian plasticity mechanisms are present in adult V1 to make those specific inputs more effective and those responses persistently stronger.

## DATA AVAILABILITY

Data will be made available upon reasonable request.

## GRANTS

This study is supported by NIH Grant R01EY002874. M.P.S. is recipient of the RPB Disney Award for Amblyopia Research.

## DISCLOSURES

No conflicts of interest, financial or otherwise, are declared by the authors.

## AUTHOR CONTRIBUTIONS

M.K. and M.P.S. conceived and designed research; M.K. and M.S.H. performed experiments; M.K., M.S.H., and M.P.S. analyzed data; M.K., M.S.H., J.A.W., and M.P.S. interpreted results of experiments; M.K. prepared figures; M.K. and M.P.S. drafted manuscript; M.K., J.A.W., and M.P.S. edited and revised manuscript; M.K., M.S.H., J.A.W., and M.P.S. approved final version of manuscript.
